# Finerenone liposome-embedded dissolving microneedles: A transcorneal platform for minimally invasive therapy of diabetic retinopathy

**DOI:** 10.1016/j.ijpx.2026.100682

**Published:** 2026-10-13

**Authors:** Weili Zhang, Lu Wang, Wen Qin, Yingrong Chen, Shenyun Fang, Zhenchao Ma, Tao Xue, Xiang Yu

**Affiliations:** a First Affiliated Hospital of Huzhou Normal University, Huzhou, Zhejiang, 313000, China; b College of Pharmacy, Shenzhen Technology University, Shenzhen, 518118, China; c Affiliated Huzhou Central Hospital, Huzhou University, Huzhou, Zhejiang, 313000, China

**Keywords:** Finerenone, Microneedle, Liposome, Diabetic retinopathy, Drug delivery

## Abstract

Diabetic retinopathy (DR) is a leading cause of vision loss characterized by retinal neovascularization and chronic inflammation. Conventional topical therapies exhibit poor corneal bioavailability and rapid precorneal clearance. Herein, we engineered a dissolvable microneedle patch incorporating finerenone-loaded liposomes (FIN-LIP@MNs) to simultaneously overcome drug solubility limitations and epithelial barrier constraints. The optimized liposomal formulation exhibited a mean diameter of 128.6 nm and high encapsulation efficiency (84.5%). PXRD and DSC further confirmed that the incorporated drug existed in an amorphous state. The MNs were characterized by drug enrichment at the needle tips, sufficient mechanical strength to penetrate the cornea, and complete dissolution within 15 min. In vivo pharmacokinetic studies showed that FIN-LIP@MNs achieved a 28.4-fold higher C_max_ and a 26.9-fold greater AUC_0~∞_ in aqueous humor compared with conventional eye drops, along with a prolonged mean residence time (6.3 h). Retinal delivery was also markedly enhanced, with C_max_ and AUC_0~∞_ increasing by 22.4-fold and 47.7-fold, respectively. In a db/db mouse model of DR, FIN-LIP@MNs restored retinal laminar architecture, rescued pericyte–endothelial homeostasis, reduced acellular capillaries by 53.8%, and markedly suppressed retinal CD31, VEGF, and inflammatory cytokines. This “liposome-in-microneedle”platform provides a minimally invasive and patient-friendly strategy for anti-angiogenic and anti-inflammatory therapy in DR.

## Graphical abstract

**Figure:**
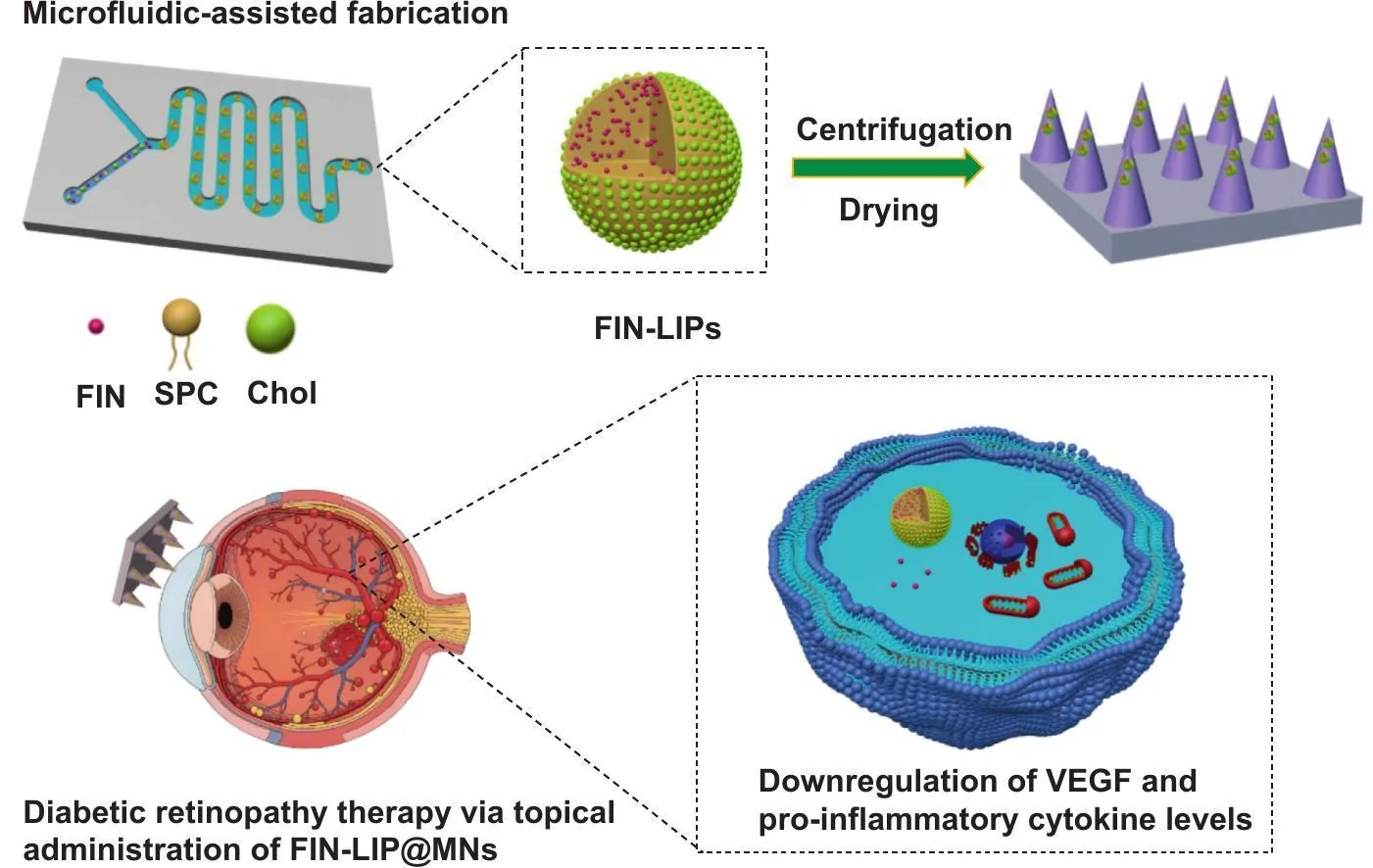

## 1 Introduction

Diabetic retinopathy (DR) is one of the most prevalent chronic microvascular complications of diabetes mellitus (Dorweiler et al., 2024; Li et al., 2023). This condition progressively impairs visual acuity and induces fundus hemorrhage. In severe cases, it may progress to retinal detachment and ultimately result in irreversible blindness (Chen et al., 2023). The pathogenesis of DR originates from the excessive generation of mitochondrial reactive oxygen species (ROS) under hyperglycemic conditions (Sun et al., 2024). ROS overproduction subsequently activates downstream signaling cascades, ultimately leading to retinal vascular damage and pathological neovascularization (Sun et al., 2024). Current therapeutic modalities include intravitreal corticosteroids, anti-angiogenic biologics, and surgical interventions such as laser photocoagulation and vitrectomy (Sinclair and Schwartz, 2024). Nevertheless, these treatment modalities are often limited by suboptimal efficacy and considerable safety risks, thereby restricting their clinical application.

Finerenone (FIN) is a non-steroidal mineralocorticoid receptor antagonist (ns-MRA) (Zhai et al., 2024). It exerts retinoprotective effects by antagonizing mineralocorticoid receptors (MR) expressed in retinal vascular endothelial cells and pericytes (Jerome et al., 2023). This mechanism attenuates oxidative stress and suppresses the release of pro-inflammatory cytokines (Lv et al., 2023). Furthermore, FIN mitigates vascular endothelial growth factor (VEGF)-mediated disruption of the blood-retinal barrier (Lv et al., 2023; Rossing et al., 2023). However, owing to its extremely poor water solubility (below 24 μg/mL) and rapid tear fluid turnover, FIN exhibits minimal corneal penetration and fails to achieve effective intraocular concentrations (Lv et al., 2023). These limitations substantially restrict its translational potential and necessitate the development of advanced delivery platforms capable of reliably maintaining therapeutic drug concentrations in the retina without compromising safety.

Nanotechnology provides an innovative strategy to overcome these drug delivery challenges (De Leo et al., 2022; Kandasamy and Maity, 2023).The bilayer structure of liposomes, composed of self-assembled phospholipids, enables the efficient encapsulation of lipophilic polyphenols through combined hydrophobic and hydrogen-bonding interactions (De Leo et al., 2022). This encapsulation strategy simultaneously enhances drug solubility, chemical stability, and mucoadhesive properties (Karami et al., 2022). However, epithelial tight junctions in the cornea present a substantial barrier to ocular drug delivery (Huang et al., 2022). These intercellular barriers markedly restrict nanocarrier permeation, thereby necessitating the integration of advanced formulation strategies to achieve effective drug delivery.

Microneedle (MN) technology has emerged as a minimally invasive approach for penetrating the corneal epithelial barrier (Garkal et al., 2022). Dissolving or hydrogel-forming MNs create micron-sized pores that enable direct stromal penetration (Garkal et al., 2022; Liu et al., 2025). This approach minimizes pain, infection risk, and permanent tissue damage (Liu et al., 2025). Consequently, the combination of microneedles with nanoparticles facilitates the delivery of drug-loaded nanocarriers into target tissues, enabling sustained drug release while preventing rapid tear dilution (Yao et al., 2024). Recently, dissolvable MNs incorporating sorafenib-loaded Soluplus nanomicelles were used for the targeted treatment of retinoblastoma (Ansari et al., 2025). Moreover, an MN system was developed by encapsulating ranibizumab-loaded PLGA nanoparticles into the needle tips for the treatment of DR (Wu et al., 2021). Although substantial progress has been achieved in ocular drug delivery platforms, the strategic integration of finerenone-encapsulated liposomes (FIN-LIPs) with MNs for DR treatment has not been comprehensively investigated.

This study reports the development of a biodegradable microneedle system incorporating liposomal finerenone (FIN-LIP@MNs) to simultaneously overcome the solubility limitations and epithelial barriers associated with conventional topical therapy. This hybrid system mechanically bypasses the corneal epithelium to enhance intrastromal drug accumulation, thereby providing superior anti-angiogenic efficacy compared with conventional eye drop formulations. This study aimed to construct, optimize, and comprehensively evaluate the biocompatibility, ocular pharmacokinetics, and therapeutic efficacy of this “liposome-in-microneedle” system in a validated mouse model of DR.

## 2 Materials and methods

### 2.1 Materials

#### 2.1.1 Animals

Male C57BL/KsJ-db/db mice (35 ± 3 g) were supplied by the Experimental Animal Center of Huzhou Central Hospital (Hangzhou, China). Animals were housed under controlled temperature (22 ± 2 °C) and humidity (55 ± 5%) with a 12 h light/dark cycle and free access to food and water. All procedures were approved by by the Ethical Committee of Huzhou Central Hospital's Academy, and performed in accordance with the ARRIVE guidelines.

### 2.2 Fabrication and optimization of FIN-LIPs

FIN-LIPs were fabricated using a microfluidic hydrodynamic focusing platform equipped with a standard T-junction chip (channel width: 200 μm; depth: 100 μm) (Mendanha et al., 2024). Briefly, finerenone (2.5 mg/mL) and varying mass ratios of soy phosphatidylcholine (SPC)/cholesterol were co-dissolved in ethanol to form the organic phase, while phosphate-buffered saline (pH 7.4) was used as the aqueous phase. The two phases were injected into the microfluidic channels through separate syringe pumps at a flow rate ratio (FRR, aqueous:organic = 3:1), with the total flow rate (TFR) maintained at 3 mL/min. This process enabled rapid nanoprecipitation and liposome self-assembly upon instantaneous ethanol dilution and solvent diffusion. The resulting colloidal suspension was immediately collected and dialyzed against distilled water for 2 h (MWCO 10 kDa) to remove residual ethanol, followed by characterization of particle size, drug loading, and encapsulation efficiency to determine the optimal FIN-LIP formulation.

### 2.3 Characterization of FIN-LIPs

#### 2.3.1 Quantification of encapsulation efficiency and drug loading capacity

A centrifugal filtration approach was utilized to separate unbound FIN from liposomes (Yuan et al., 2025). Specifically, 1.0 mL aliquots of FIN-LIPs were introduced into Amicon® Ultra-4 centrifugal concentrators (30 kDa molecular weight cutoff) and spun at 5000 ×*g* for 10 min at 4 °C. The filtrate, enriched with free FIN, was collected for subsequent analysis. A reversed-phase HPLC method was developed for the quantitative determination of FIN via HPLC system (Agilent1260 Infinity II, USA) (Marie et al., 2025). Separation was performed on a C18 column (150 × 4.6 mm, 3.5 μm) with an isocratic mobile phase consisting of acetonitrile and 10 mM ammonium acetate buffer (pH 3.5, 70:30, *v*/v) at a flow rate of 1.0 mL/min, column temperature of 35 °C, and UV detection at 254 nm. The filtrate sample was filtered through a 0.22 μm membrane, and then injected at a volume of 20 μL. Encapsulation efficiency (EE, %) and Drug loading capacity (DL, %) were calculated according to the following equations:

(1) $$\mathrm{EE}=\frac{W_{\mathrm{total}}-W_{\mathrm{free}}}{W_{\mathrm{total}}}\times 100\%$$

(2) $$\mathrm{DL}=\frac{W_{\mathrm{total}}-W_{\mathrm{free}}}{W_{\mathrm{total}}-W_{\mathrm{free}}+W_{p}}\times 100\%$$

*W*_*total*_ represents the aggregate mass of FIN incorporated within the liposomal suspension, whereas *W*_*free*_ indicates the quantity quantified in the permeate fraction. *W*_*p*_ denotes the combined weight of SPC, cholesterol, and finerenone initially employed. All determinations were conducted in three independent replicates, with numerical results reported as arithmetic mean ± standard deviation (SD).

#### 2.3.2 Determination of particle size and zeta potential of FIN-LIPs

A Malvern Zetasizer Nano ZS90 (Malvern Instruments, UK) was utilized to characterize the mean vesicle diameter and surface charge (ζ-potential) of FIN-LIPs at 25 °C. The formulations were first diluted to 0.1% (*w*/*v*) with sterile-filtered PBS (pH 7.4, 0.22 μm) and subsequently allowed to stabilize for 120 s prior to data acquisition.

#### 2.3.3 Microscopic morphology evaluation of FIN-LIPs

The ultrastructural morphology of FIN-LIPs was examined using transmission electron microscopy (TEM). Briefly, FIN-LIPs were diluted to an appropriate concentration (0.05 mg/mL) with deionized water. 5 μL of the diluted sample was carefully deposited onto a 300-mesh carbon-coated copper grid and allowed to adsorb for 3 min. Excess liquid was gently blotted away with filter paper. Subsequently, the specimens were negatively stained with 2% uranyl acetate solution for 60 s, followed by air-drying at ambient temperature for 30 min. Imaging was performed on a JEM-1400 Flash transmission electron microscope (JEOL, Tokyo, Japan).

#### 2.3.4 Diffractometric assessment of crystallinity in FIN-LIPs

Crystallographic analysis was performed using a Bruker D8 Advance diffractometer operating with Cu Kα radiation at a wavelength of 1.5406 Å. Prior to measurement, lyophilized specimens (FIN-LIPs, pure drug, blank liposomes, and their physical admixtures) were comminuted in an agate mortar and packed into zero-background silicon holders. The angular scanning ranged from 5° to 60° (2θ) with a step width of 0.02° and a step time of 0.5 s.

#### 2.3.5 Thermal characterization of FIN-LIPs by differential scanning calorimetry

Differential scanning calorimetry (Mettler-Toledo DSC 3+) was employed to assess the thermal behavior of finerenone formulations. The instrument was verified using indium calibration standards prior to analysis. Samples (~5 mg), including FIN-LIPs, free FIN, blank liposomes, and their physical combinations, were placed in 40 μL aluminum sample pans with pierced covers and hermetically sealed; an empty pan served as reference. After equilibrating at 25 °C, specimens were heated from 30 to 300 °C at 10 °C/min. Thermograms were analyzed for phase transition temperatures (onset and peak) and enthalpy changes using dedicated STARE software.

### 2.4 Fabrication of FIN-LIP@MNs

A centrifugation-assisted molding protocol was employed to fabricate FIN-LIP-embedded dissolvable MNs. A commercially available PDMS conical MN mold (Xiamen Qipu Medical Technology Co., Ltd., China) was employed, featuring 10 × 10 conical cavities (600 μm depth, 300 μm base width) with 650 μm centre-to-centre spacing. The 9 mm × 9 mm patch footprint achieved ~123 needles/cm^2^ for uniform drug distribution. Lyophilized liposomal powder was first reconstituted in deionized water to prepare a concentrated stock suspension, followed by incorporation into a polymeric matrix comprising PVP K90 at optimized proportions to obtain a clear and viscous precursor. The blend was subjected to vacuum treatment (−0.1 MPa, 10 min) to remove entrapped gaseous inclusions that could compromise MN mechanical properties. The degassed formulation (150 μL) was transferred onto PDMS negative molds and centrifuged at 2500 ×*g* for 5 min at ambient temperature to ensure complete penetration into microcavity features. Excess material was carefully scraped away using a sterile blade, and the filled templates were air-dried until the microneedle shafts achieved structural rigidity. A supporting base matrix was subsequently constructed by depositing 150 μL of aqueous PVP K90 solution (15% *w*/w) onto the mold surface and permitting spontaneous desiccation at room temperature, yielding a transparent, robust film (~280 μm). The obtained patches were custom-trimmed into a 6 × 6 microneedle array configuration (600 μm needle height, 3 × 3 mm^2^ array area) to fit the murine cornea (~2.5 mm in diameter) and then preserved in a desiccated environment (RH ≤ 20%). The drug content localized within the needle (M_n_) versus the basal layer (M_b_) was determined following physical dissection with a scalpel.

### 2.5 Characterization of FIN-LIP@MNs

#### 2.5.1 Ultrastructural examination of FIN-LIP@MNs

FIN-LIP@MNs were affixed to aluminum stubs with conductive carbon tape and gold-sputtered prior to imaging. Field-emission scanning electron microscopy (FE-SEM, Verios 5 XHR, USA) was conducted at 2 kV accelerating voltage and 100 pA beam current, capturing secondary electron images at 45° tilt angles to assess microneedle geometry. Parallel fluorescence studies employed FITC-encapsulated liposomes prepared as aforementioned, which were incorporated into microneedle arrays (FITC-LIP@MNs). Arrays were mounted on glass slides and visualized using fluorescence microscopy (FM-200, Puguang, Shanghai, China; λ_ex_ 488 nm, λ_em_ 520 ± 20 nm) to confirm uniform drug distribution within the polymeric matrix.

#### 2.5.2 Mechanical performance evaluation of FIN-LIP@MNs

Uniaxial compression testing was performed on an Instron 68SC-05 micro-mechanical tester. Individual FIN-LIP@MN patches were fixated onto a stainless-steel stage via double-sided adhesive tape. A flat-faced cylindrical indenter (2 mm diameter) was then pressed onto the needle tips at a displacement rate of 0.1 mm s^−1^ while recording force data at 1 kHz. The assay involved incremental loading up to 50 mN maximum force. Compressive stiffness was calculated from the slope of the linear portion of the force-displacement curve. The slope of the initial linear region represents the compression stiffness, which was then converted into compressive modulus (*E*) according to the microneedle geometry using the formula *E* = *k* × L / A, where *k* is stiffness, L is needle height, and A is the base cross-sectional area.

#### 2.5.3 Ocular insertion evaluation of FIN-LIP@MNs

Male C57BL/KsJ-db/db mice (35 ± 3 g) were anesthetized, and the right eye was gently exteriorized using a sterile eyelid retractor. A single FIN-LIP@MN patch was manually grasped with self-locking forceps, positioned perpendicular to the central cornea, and pressed against the ocular surface for 30 s. Immediately following application, the treated corneal tissue was surgically excised and subjected to histological processing: fixation in 10% neutral-buffered formalin (24 h), cryoprotection in optimal cutting temperature (OCT) compound, flash-freezing in liquid nitrogen, and cryosectioning at 8 μm thickness. Sections were mounted onto glass slides, subjected to hematoxylin-eosin staining, and examined via bright-field microscopy (FM-200, Shanghai Puguang) to evaluate microneedle penetration depth, epithelial layer disruption, and stromal infiltration.

#### 2.5.4 Dissolution kinetics of FIN-LIP@MNs in corneal tissue

Male C57BL/KsJ-db/db mice (35 ± 3 g) were housed under temperature-regulated conditions. Prior to the intervention, topical anesthesia of the right eye was induced by lidocaine hydrochloride instillation. FIN-LIP@MNs were subsequently positioned at the center of the corneal surface and maintained in contact for predetermined time intervals (0 min, 5 min and 10 min) after which the patches were carefully removed. Progressive matrix dissolution was monitored in realtime using a stereomicroscope (FM-200, Shanghai Puguang).

#### 2.5.5 In vitro assessment of FIN-LIP@ MNs

The release profile of FIN-LIP@MNs was investigated using eye-simulated fluid (ESF: 6.4 g L^−1^ NaCl, 1.0 g L^−1^ NaHCO₃, 0.5 g L^−1^ CaCl₂·2H₂O, and 0.2 g L^−1^MgCl₂·6H₂O; pH 7.4). Individual patches (containing ~105 μg FIN) were immersed in 25 mL of pre-warmed ESF (34 ± 1 °C) in 50 mL Erlenmeyer flasks and agitated at 100 rpm in a temperature-controlled shaker. At predetermined time points (0.25, 0.5, 0.75, 1, 1.5, and 2 h), 1 mL aliquots were collected and immediately replaced with an equal volume of fresh, thermally equilibrated ESF. Following filtration through 0.22 μm PVDF membranes and 1:1 dilution with acetonitrile, the drug concentration was quantified using the HPLC method described in Section 2.4.1. The cumulative release percentage (*Q*_*t*_) was calculated relative to the initial FIN loading of each patch and expressed as a function of time. Mathematical modeling of the release kinetics of FIN-LIP@MNs was performed by fitting the fractional drug release data to established kinetic equations.

First-order model,

(3) $$\ln\left(1-Q_{t}\right)=\mathrm{kt}$$

Zero-order model,

(4) $$Q_{t}=\mathrm{kt}$$

Higuchi model,

(5) $$Q_{t}=kt^{\frac{1}{2}}$$

Ritger–Peppasmodel,

(6) $$Q_{t}=kt^{n}$$

where *Q*_*t*_ is defined as the cumulative release fraction at specific time t, with k denoting the release velocity constant for FIN from the FIN-LIP@MNs.

#### 2.5.6 Ex vivo characterization of the transcorneal transport behavior of FIN-LIP@MNs

Freshly isolated C57BL/KsJ-db/db mice corneas (0.191 cm^2^) were mounted in Franz diffusion cells maintained at 32 °C to evaluate transcorneal penetration. The epithelial surface was treated with either FIN-LIP@MNs or aqueous FIN suspensions (~105 μg) through direct contact of the MN tips or uniform spreading (200 μL for the control group), secured using magnetic clamps. The receptor chamber contained 5 mL of eye-simulated fluid supplemented with 0.5% (*w*/*v*) Tween 80 and was agitated at 300 rpm to maintain sink conditions. Serial sampling (300 μL) was performed with immediate isovolumetric replacement. FIN concentrations were quantified using the HPLC method described in Section 2.4.1. (Marie et al., 2025). Permeation profiles were constructed and steady-state flux calculated by normalizing the linear segment slopes to the diffusion surface area.

### 2.6 In vivo safety evaluation of FIN-LIP@MNs

A total of 12 male C57BL/KsJ-db/db mice (weighing 35 ± 3 g) were randomly assigned to the FIN-LIP@MN treatment group and the untreated group (Baseline control, *n* = 6 per group). Under 3% isoflurane anesthesia, corneal barrier integrity was evaluated immediately after 30 s of MN application by transepidermal water loss measurement combined with fluorescein strip testing. After daily MN administration for 7 consecutive days, all mice were euthanized under anesthesia. The harvested tissues, including the retina, cornea, heart, liver, spleen, lung, and kidney, were fixed in 4% paraformaldehyde for 48 h, dehydrated through a graded ethanol series, cleared in xylene, and embedded in paraffin. Sections of 5 μm thickness were prepared, dewaxed in xylene, rehydrated through descending ethanol concentrations, and stained with hematoxylin for 3 min followed by eosin for 3 min. After dehydration in graded ethanol and clearing in xylene, the sections were mounted with neutral balsam and examined under a light microscope.

### 2.7 Ocular pharmacokinetic evaluation of FIN-LIP@MNs

Male C57BL/KsJ-db/db mice (35 ± 3 g) were randomly allocated into two parallel cohorts (*n* = 66 per group) to receive either a single administration of FIN-LIP@MN or 10 μL of FIN suspension (equivalent dose, ~105 μg). Sequential sampling (*n* = 6 per time-point) occurred at 0.25, 0.50, 1.0, 1.5, 3.0, 5.0, 7.0, 10, 16, 20 and 24 h. Following CO_2_ euthanasia and immediate eye excision, aqueous humor 5 μL) was harvested via anterior chamber puncture (30 G needle), diluted with ice-cold acetonitrile (1:20, *v*/v), and centrifuged (12,000 ×*g*, 10 min, 4 °C). Supernatants were maintained at −80 °C prior to FIN quantification. For retinal analysis,the retinas were carefully dissected from the posterior segment under a stereomicroscope (Leica, Germany), snap-frozen in liquid nitrogen, lyophilized to constant weight, and precisely weighed. Each dried retinal sample was mechanically homogenized in 9 volumes (*w*/*v*) of ice-cold acetonitrile containing the internal standard ([^2^H_5_]-FIN, 50 ng/mL) using a high-speed tissue homogenizer (Precellys, Bertin Technologies, France) at 6000 rpm for 30 s. The homogenate was centrifuged at 12,000 ×*g* for 15 min at 4 °C, and the resulting supernatant was transferred to a clean glass tube and evaporated to dryness under a gentle nitrogen stream at 40 °C. The residue was reconstituted in 100 μL of mobile phase, vortex-mixed for 30 s, and centrifuged at 15,000 ×*g* for 10 min at 4 °C.

A validated high-performance liquid chromatography–tandem mass spectrometry (HPLC–MS/MS) assay was developed for the quantitative determination of FIN (Rohde et al., 2021). [^2^H_5_]-FIN was selected as the internal standard (ISD). Chromatographic analysis was performed on a Vanquish Flex UHPLC system coupled with an Orbitrap Exploris 120 mass spectrometer (Thermo Fisher Scientific, USA) using an Acquity UPLC BEH C18 column (1.7 μm, 20 × 2.1 mm; Waters, Milford, MA, USA). Isocratic elution was carried out with a mobile phase of 0.1% formic acid (A) and acetonitrile (B) (90:10, *v*/v) at a flow rate of 0.1 mL/min for a total run time of 3 min. Detection was conducted by positive-ion MRM (*m*/*z* 379.1/218.1 for FIN; *m*/*z* 384.1/218.1 for the ISTD). Pharmacokinetic parameters (C_max_, T_max_, AUC, MRT) were generated from concentration-time profiles using non-compartmental analysis in WinNonlin 8.3.

### 2.8 Pharmacodynamic study

#### 2.8.1 Animal modeling and grouping

Male C57BL/KsJ-db/db mice (35 ± 3 g) were used as a spontaneous type 2 diabetic retinopathy model. Animals were housed under specific pathogen-free (SPF) conditions at 22 ± 2 °C and 50 ± 10% relative humidity under a 12 h light/dark cycle, with free access to a high-fat diet (60% kcal from fat) and sterilized water. Following a 7-day acclimatization period, fasting blood glucose (FBG) levels were measured via tail vein puncture. Successful diabetes induction was defined as FBG ≥ 11.1 mmol/L on two consecutive measurements, accompanied by elevated glycated hemoglobin (HbA1c ≥ 6.5%). The diabetic state was maintained for 12 weeks to induce moderate-to-severe non-proliferative diabetic retinopathy (NPDR), with disease confirmation established by electroretinography showing a > 30% reduction in b-wave amplitude, consistent with microaneurysm formation and early neurovascular impairment. Male C57BL/KsJ-db/db mice without DR were included as baseline control group (*n* = 9). Confirmed DR mice were randomly assigned to four groups nd received daily administration for 7 days (*n* = 9 per group): untreated control, liposome-loaded MNs without drug encapsulation (blank MNs), FIN suspension eye drops (10 μL, 10.5 mg/mL, qd), and FIN-LIP@MNs (105 μg FIN/array, qd).

#### 2.8.2 Hematoxylin and eosin staining

The aforementioned mice were deeply anesthetized and euthanized, and the eyeballs were immediately enucleated after one week of treatment. Specimens were fixed in 4% paraformaldehyde for 48 h, dehydrated through graded ethanol, cleared in xylene, and embedded in paraffin along the optic axis. Coronal serial sections (4 μm thick) were obtained at 100 μm intervals. Following deparaffinization and rehydration, sections were stained with hematoxylin solution (8–10 min), differentiated with acid alcohol, blueing in running water, counterstained with 0.5% eosin Y (2 min), dehydrated, cleared in xylene, and mounted with neutral balsam. Microaneurysms at the junction of the inner nuclear layer (INL) and outer plexiform layer (OPL) and capillary lumen diameters were examined under light microscopy.

#### 2.8.3 Periodic acid-schiff staining

The aforementioned sections were oxidized in 0.5% periodic acid solution for 10 min, followed by thorough rinsing with distilled water. Subsequently, the sections were incubated in Schiff reagent for 15 min at room temperature in the dark. After staining, the sections were washed under running tap water for 10 min to allow the magenta coloration to develop and stabilize. Nuclei were lightly counterstained with hematoxylin (10–20 s), differentiated in 1% acid alcohol, and blued under running water. The sections were then dehydrated through graded ethanol, cleared in xylene, and mounted with neutral balsam. PAS-positive structures, including vascular basement membranes and microaneurysms, appeared magenta. Retinal microvascular morphology, acellular capillaries, and the pericyte-to-endothelial cell ratio were examined under light microscopy. Quantitative analysis was performed using ImageJ software.

#### 2.8.4 Immunofluorescence staining and quantification of CD31 in retinal tissue

Retinal sections (10 μm) were fixed in 4% paraformaldehyde, permeabilized with 0.3% Triton X-100, and blocked with 5% BSA for 1 h. Samples were incubated overnight at 4 °C with rat anti-mouse CD31 antibody (1:200), followed by incubation with Alexa Fluor 488-conjugated secondary antibody (1:500) for 2 h at room temperature. Nuclei were counterstained with DAPI. Images were acquired using a fluorescence microscope and analyzed using ImageJ software to determine the mean optical density (MOD) and the percentage of VEGF-positive area relative to the total retinal area.

#### 2.8.5 Immunohistochemistry-based quantification of VEGF levels in retinal tissue

Retinal sections (4 μm) were deparaffinized in xylene and rehydrated through graded ethanol series, followed by antigen retrieval in sodium citrate buffer (pH 6.0) at 95 °C for 20 min. Endogenous peroxidase activity was quenched with 3% H2O2 for 10 min, and nonspecific binding sites were blocked with 5% normal goat serum for 30 min at room temperature. Sections were incubated overnight at 4 °C with rabbit polyclonal anti-VEGF antibody (1:200 dilution), followed by incubation with HRP-conjugated secondary antibody (1:500) for 1 h at 37 °C. Immunoreactivity was visualized using DAB chromogen, followed by hematoxylin counterstaining, dehydration, and mounting with neutral balsam. VEGF immunohistochemical sections were digitized under identical illumination conditions. Semiquantitative analysis was performed using Image J software by measuring the mean optical density (MOD) of DAB-positive brown staining within the total retinal area.

#### 2.8.6 Assessment of inflammation-related protein expression in retinal tissue

Retinal tissues were homogenized in RIPA buffer for protein extraction. Total protein concentrations were determined using a BCA assay kit. Subsequently, retinal lysates were subjected to enzyme-linked immunosorbent assay (ELISA) for the quantitative measurement of inflammatory cytokines (IL-6, TNF-α, and ICAM-1) according to the manufacturer's protocols. Cytokine concentrations were calculated based on standard curves.

### 2.9 Statistical analysis

All experimental data were analyzed statistically using SPSS 16.0 software package. Comparisons among multiple groups were performed using one-way analysis of variance (ANOVA).

## 3 Results and discussion

### 3.1 Optimal formulations for fabrication of FIN-LIPs

FIN-LIPs were fabricated using a microfluidic-assisted nanoprecipitation strategy, followed by systematic optimization of the SPC/cholesterol mass ratio and drug-to-SPC ratio to maximize encapsulation efficiency (EE) while maintaining nanoscale particle size. Initial optimization focused on the drug-to-SPC mass ratio. Under a fixed lipid composition (SPC/Chol = 4:1) with FIN dissolved in ethanol at 2.5 mg/mL, a drug-to-SPC ratio of 1:8 (*w*/w) achieved an optimal balance between encapsulation efficiency (84.5 ± 1.5%) and drug loading capacity (7.7 ± 0.6%, Table 1). Higher ratios (>1:5) resulted in drug precipitation and reduced encapsulation efficiency due to saturation of the hydrophobic domains within the lipid bilayer, whereas lower ratios compromised the absolute drug payload required for therapeutic efficacy. Concurrently, the SPC/Chol mass ratio was evaluated. Progressive incorporation of cholesterol up to 20% (SPC/Chol = 4:1, *w*/w) significantly increased encapsulation efficiency from 71.5% to 84.5% by enhancing the hydrophobic core volume and reducing membrane permeability (Table 2). However, excessive cholesterol incorporation (>30%) induced excessive membrane rigidity, resulting in increased particle size (>200 nm) and compromised colloidal stability.

**Table 1:** Effect of drug/SPC ratio on FIN-LIP encapsulation performance under a fixed lipid composition (SPC/Chol = 4:1) (*n* = 3).

Drug/SPC (*w*/w)	EE (%)	DL (%)	PS (nm)	Precipitation
1:4	67.1 ± 1.8	11.1 ± 0.7	115.3 ± 9.1	Yes (visible crystals)
1:8	84.5 ± 1.5	7.7 ± 0.2	127.8 ± 3.1	No
1:12	89.6 ± 2.9	5.6 ± 0.8	146.5 ± 5.6	No
1:16	91.2 ± 1.5	4.3 ± 0.5	155.1 ± 4.8	No

**Table 2:** Effect of SPC/Chol mass ratio on FIN-LIP encapsulation performance under a fixed drug-to-SPC ratio (FIN/SPC = 1:8, *n* = 3).

SPC/Chol (w/w)	EE (%)	DL (%)	PS (nm)	Precipitation
8:1	71.5 ± 5.7	7.2 ± 0.2	142.5 ± 5.1	No
6:1	76.9 ± 3.7	7.4 ± 0.1	136.8 ± 4.9	No
4:1	84.5 ± 1.5	7.7 ± 0.2	127.8 ± 3.1	No
2:1	80.6 ± 1.8	6.2 ± 0.5	201.4 ± 9.7	No

The optimized formulation (FIN at 2.5 mg/mL in ethanol, drug-to-SPC ratio of 1:8, and SPC/Chol ratio of 4:1) was obtained, with an encapsulation efficiency of 84.5% and a drug loading of 7.7%. Moreover, the optimized FIN-LIP exhibited a characteristic bluish opalescence and monodisperse spherical morphology, with a mean hydrodynamic diameter of 128.6 ± 4.2 nm (PDI = 0.19) and a zeta potential of −21.3 ± 1.5 mV. These properties collectively indicated favorable colloidal stability (Fig. 1A, B, and C).

**Fig. 1:**
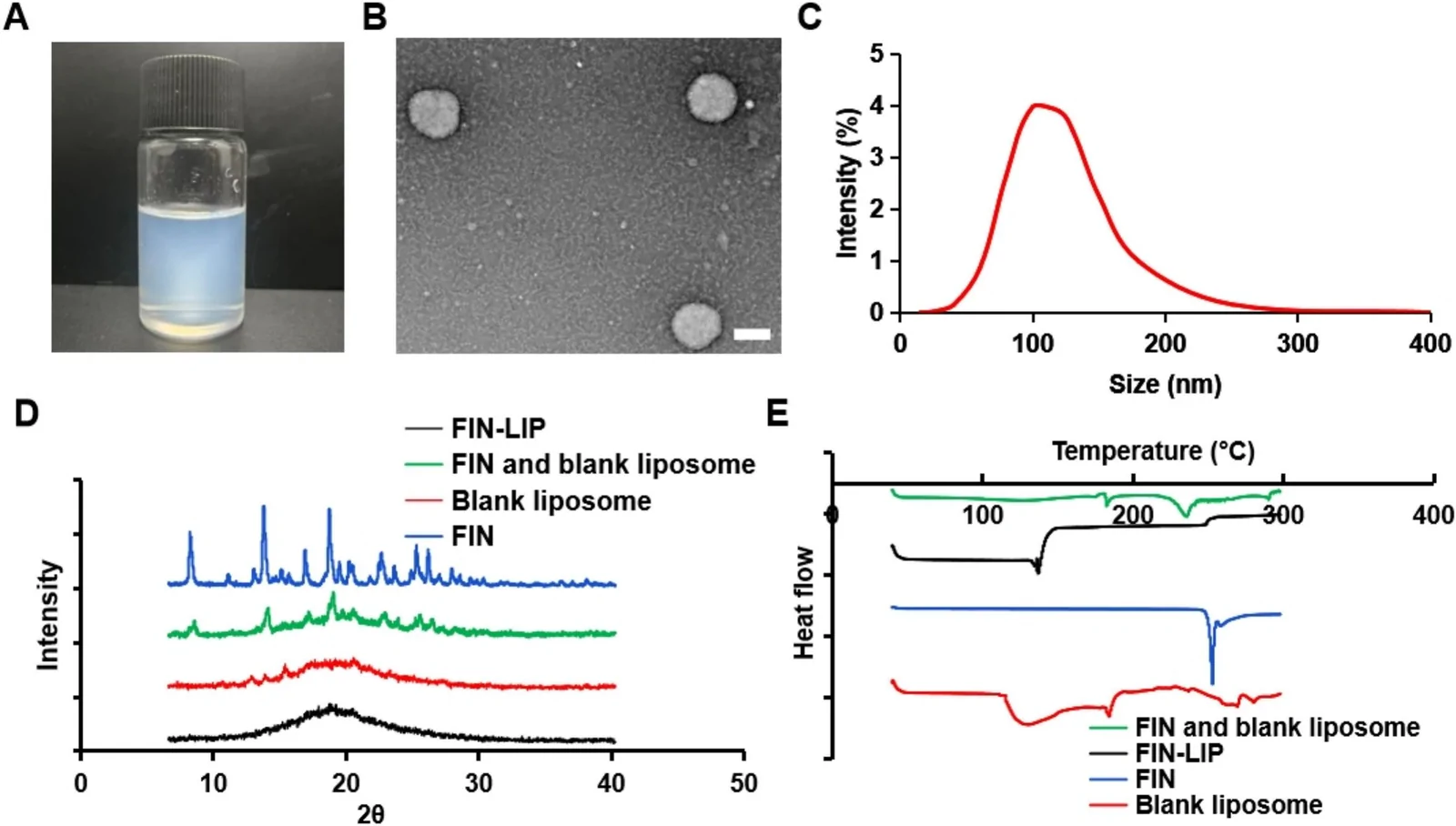
Physicochemical characterization of the optimized FIN-LIP formulation. (A) Digital photograph of the FIN-LIP aqueous dispersion; (B) Representative TEM image of FIN-LIPs (scale bar = 100 nm); (C) Hydrodynamic diameter distribution of FIN-LIPs; (D) X-ray diffraction (XRD) pattern of FIN-LIPs; (E) Differential scanning calorimetry (DSC) thermogram of FIN-LIPs.

### 3.2 Solid-state properties of FIN-LIPs from PXRD analysis

Powder X-ray diffraction patterns revealed distinct solid-state characteristics among FIN, blank liposomes, and FIN-LIPs (Fig. 1D). Free FIN exhibited sharp diffraction peaks at 2θ values of 8.5°, 12.3°, 16.8°, 19.2°, 21.5°, and 24.6°, indicating a highly crystalline structure with long-range molecular order. In contrast, blank liposomes (SPC/Chol = 4:1) displayed a broad diffuse halo centered at 2θ = 13–23°, confirming the amorphous nature of the lipid matrix. Notably, the optimized FIN-LIPs showed complete disappearance of the characteristic crystalline peaks of the free drug. Specifically, the presence of a broad and featureless amorphous halo suggested successful encapsulation of FIN within the lipid bilayer in a molecularly dispersed state. To exclude dilution artifacts, a physical mixture of free FIN and blank liposomes (1:10, w/w) was subjected to PXRD analysis. This sample retained all sharp diffraction peaks characteristic of crystalline FIN, which were distinctly superimposed on the broad amorphous halo of the lipid matrix. Therefore, the amorphous transformation observed in the optimized FIN-LIPs was attributed to genuine drug entrapment within the vesicular core rather than simple mechanical mixing.

### 3.3 Thermotropic properties of of FIN-LIPs from DSC analysis

Consistent with the PXRD findings, differential scanning calorimetry (DSC) analysis further confirmed the amorphous transformation of FIN following liposomal encapsulation. Free FIN exhibited a sharp endothermic melting peak at approximately 256 °C (Fig. 1E), indicative of its crystalline lattice structure, whereas blank liposomes displayed a characteristic broad phase transition at ~122 °C corresponding to the gel-to-liquid crystalline transition of the lipid bilayer. Notably, the optimized FIN-LIPs completely lacked the characteristic drug melting endotherm throughout the thermal range of 30–300 °C. To exclude the possibility that the loss of crystallinity resulted merely from drug dilution rather than true encapsulation, a physical mixture of free FIN and blank liposomes (1:10, w/w) was analyzed and clearly retained the sharp endothermic peak at 256 °C superimposed on the lipid baseline. These findings confirmed that the thermal disappearance of FIN in the loaded formulation resulted from genuine molecular encapsulation within the hydrophobic core rather than simple compositional dilution. The combined evidence from PXRD (loss of crystallinity) and DSC (absence of melting enthalpy) collectively demonstrated that FIN existed in a molecularly dispersed or amorphous state within the liposomal bilayer. Such solid-state modification is conducive to enhanced apparent solubility and sustained-release behavior.

### 3.4 The optimized formulation of FIN-LIP@MNs

Dissolvable MNs have gained considerable attention as an innovative platform for ocular drug delivery (Kim et al., 2022). Owing to their residue-free bioresorbable matrices, scalable production, high delivery efficiency, and improved patient acceptability (Ming et al., 2026), dissolvable MNs were developed in this study to facilitate FIN-LIP delivery to retinal tissues.

PVP K90, a pharmaceutically acceptable excipient with established biocompatibility and hydrosolubility, was used as the MN matrix material in this study (Jian et al., 2025). Initial solubility assessments demonstrated that unencapsulated FIN at 0.1% (*w*/*v*) failed to dissolve in aqueous PVP solutions at 15% (*w*/*v*), resulting in a heterogeneous suspension (Fig. 2A). In contrast, FIN-LIPs at 1.25% (*w*/*v*), corresponding to a 0.1% FIN dose, were uniformly within the same hydrogel matrix. These findings demonstrated that encapsulation within phospholipid vesicles effectively overcame the aqueous solubility limitations of FIN, thereby significantly improving the drug-loading capacity of the MN formulation.

**Fig. 2:**
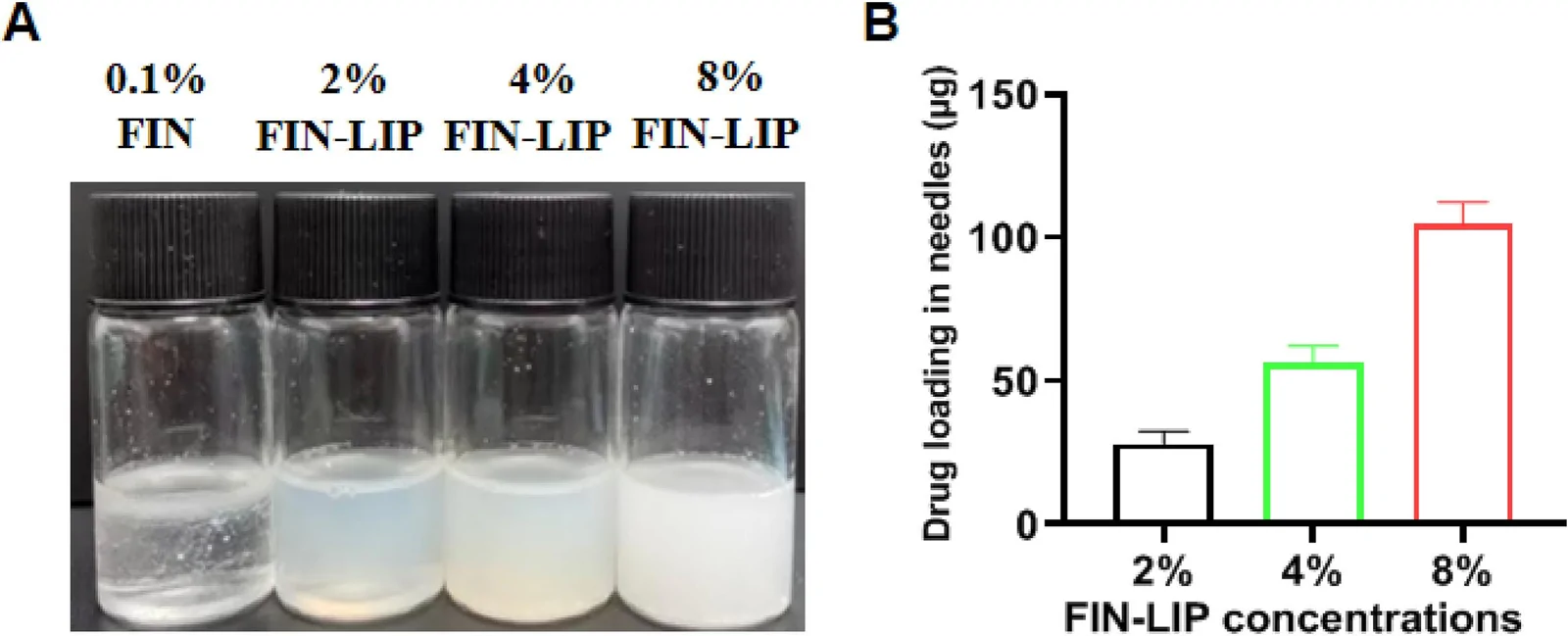
Compositional optimization of the FIN-LIP@MN formulation. (A) Aqueous solubility profiling of native FIN and liposomal FIN within 15% PVP solutions; (B) Drug payload distribution at the needle apices across varying FIN-LIP concentrations.

The stratum corneum can be effectively penetrated by MNs fabricated from a 15% (*w*/w) aqueous PVP solution; however, the mechanical strength required for corneal insertion is substantially reduced (Ching et al., 2025; Than et al., 2018). Previous studies have demonstrated that MNs fabricated from a 15% (*w*/w) aqueous PVP solution can effectively penetrate the stratum corneum (Ma et al., 2024). Accordingly, this concentration was selected as the optimal matrix composition for FIN-LIP@MNs in the present study. Successful transcorneal delivery of nanomedicines remains a major challenge, necessitating encapsulation of therapeutic agents within the needle tips rather than the supporting base (Arafa et al., 2020; Liu et al., 2025). Therefore, the MN tips must contain a sufficient drug payload to achieve the therapeutic threshold. In mice, the recommended intraperitoneal dose of FIN for suppressing retinal neovascularization is 100 μg (Jerome et al., 2023). In the present study, MNs were prepared through systematic optimization of the FIN-LIP concentration within the matrix. When the FIN-LIP concentration reached 8% (*w*/*v*), the FIN payload localized in the MN tips was approximately 105 μg (Fig. 2B), thereby achieving the required therapeutic dose. Based on these findings, the optimized formulation of FIN-LIP@MNs consisted of 8% (*w*/*v*) FIN-LIPs dispersed in a 15% (*w*/w) aqueous PVP solution.

The physicochemical stability of FIN-LIPs within the microneedle matrix was maintained throughout the 6-month storage duration. The reconstituted liposomes exhibited intact morphology and consistent particle sizes of approximately 130 nm (Fig. S1A), while encapsulation efficiencies at 0, 3, and 6 months were 82.9 ± 4.1%, 86.0 ± 3.9%, and 82.3 ± 3.2%, respectively. No statistically significant differences were observed across the tested time points (*p* > 0.05, Fig. S1B), confirming that the microneedle formulation effectively stabilizes FIN-LIPs.

### 3.5 Characteristics of FIN-LIP@MNs

#### 3.5.1 Morphology of FIN-LIP@MNs

The fabricated FIN-LIP@MNs exhibited a precisely engineered conical microneedle array with tip diameters of approximately 16 μm, axial heights of 605 μm, and basal widths of 300 μm (Fig. 3A). To visualize intravesicular drug distribution within the MNs, FITC-loaded liposomes were synthesized using the same protocol as that used for FIN-LIP preparation and subsequently incorporated into the dissolvable matrix. Fluorescence micrographs revealed distinct accumulation of these fluorophore-labeled carriers at the needle tips, with fluorescence intensity markedly higher than that observed in the supporting base layer (Fig. 3B and C). Quantitative analysis further confirmed this distribution pattern. The apical regions contained 104.8 ± 7.1 μg of FIN, representing a 5.6-fold enrichment compared with the basal layer, which contained only 18.6 ± 4.3 μg (Fig. 3D). This selective tip loading demonstrates that FIN-LIP@MNs achieve site-specific drug accumulation at the tissue penetration interface, thereby maximizing local bioavailability.

**Fig. 3:**
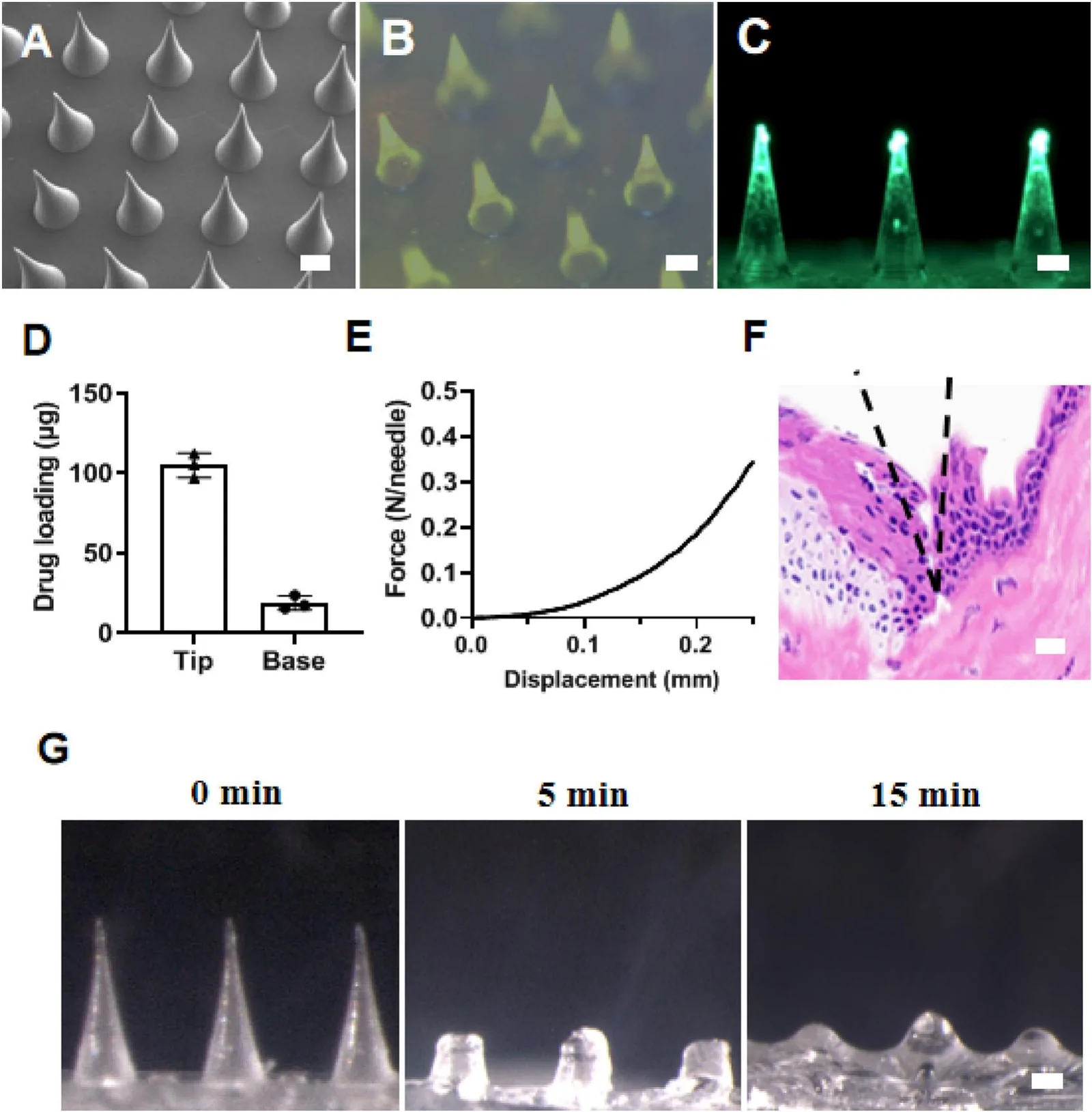
Characteristics of FIN-LIP@MNs. (A) Representative SEM images illustrating the morphological features of FIN-LIP@MNs (scale bar = 200 μm); (B) Stereomicroscopic images of FITC-LIP@MNs (scale bar = 200 μm); (C) Fluorescence imaging of FITC-LIP@MNs demonstrating uniform liposome distribution (scale bar = 200 μm); (D) Drug payload distribution throughout the MNs; (E) Force-displacement curve revealing the mechanical insertion properties; (F) Histological examination of corneal sections following FIN-LIP@MN insertion via H&E staining (scale bar = 10 μm); (G) Time-dependent dissolution profile of FIN-LIP@MNs following intracorneal implantation in rats (scale bar = 200 μm).

#### 3.5.2 Mechanical properties of FIN-LIP@MNs

The ability of MNs to withstand insertion forces without structural failure is essential for effective transcorneal drug delivery (Aghaei et al., 2026; Elhabal et al., 2025). Axial compression testing was performed to evaluate the mechanical strength of FIN-LIP@MNs (Fig. 3E). The fabricated MNs demonstrated a compressive strength of 36.4 MPa, substantially exceeding the threshold of 17.7 MPa required for reliable corneal penetration (Than et al., 2018). Given that the ocular surface presents significantly lower mechanical resistance than the skin, these findings indicate that the FIN-LIP@MN system possesses sufficient mechanical robustness to enable consistent corneal insertion while preserving tissue integrity.

#### 3.5.3 Transcorneal insertion characteristics of FIN-LIP@MNs

The corneal epithelium serves as a major physical barrier to foreign particle penetration, with a thickness of approximately 50–60 μm (Tang et al., 2022). Precise control of insertion depth is critical for optimizing drug deposition within retinal tissues (Toprangkobsin et al., 2022). The fabricated FIN-LIP@MNs exhibited a penetration depth of approximately 78 μm in corneal tissue (Fig. 3F). This depth effectively traversed the epithelial barrier while preserving the structural integrity of the underlying stroma. These findings demonstrate the considerable potential of this MN system to enhance drug delivery efficiency and therapeutic efficacy.

#### 3.5.4 Rapid bioresorption of FIN-LIP@MNs following corneal insertion

The dissolution kinetics of MNs represent a critical prerequisite for effective transcorneal administration (Wu et al., 2022). Following corneal application, the FIN-LIP@MN tips exhibited extensive dissolution within 5 min (Fig. 3G), with complete bioresorption observed within 15 min. These rapid dissolution properties further support the suitability of this system for efficient transcorneal drug delivery.

#### 3.5.5 In vitro drug release profiles of FIN-LIP@MNs

Rapid drug release from MNs markedly facilitates transcorneal administration (Roy et al., 2022). FIN-LIP@MNs exhibited pronounced burst-release behavior, achieving approximately 52% payload release within 0.5 h. The cumulative drug release reached approximately 94% at 1.5 h (Fig. 4A). Kinetic analysis demonstrated that the release profile conformed to the First-Order model (R^2^ = 0.98, Table 3), indicating that the drug release rate was directly proportional to the residual drug content within FIN-LIP@MNs.

**Fig. 4:**
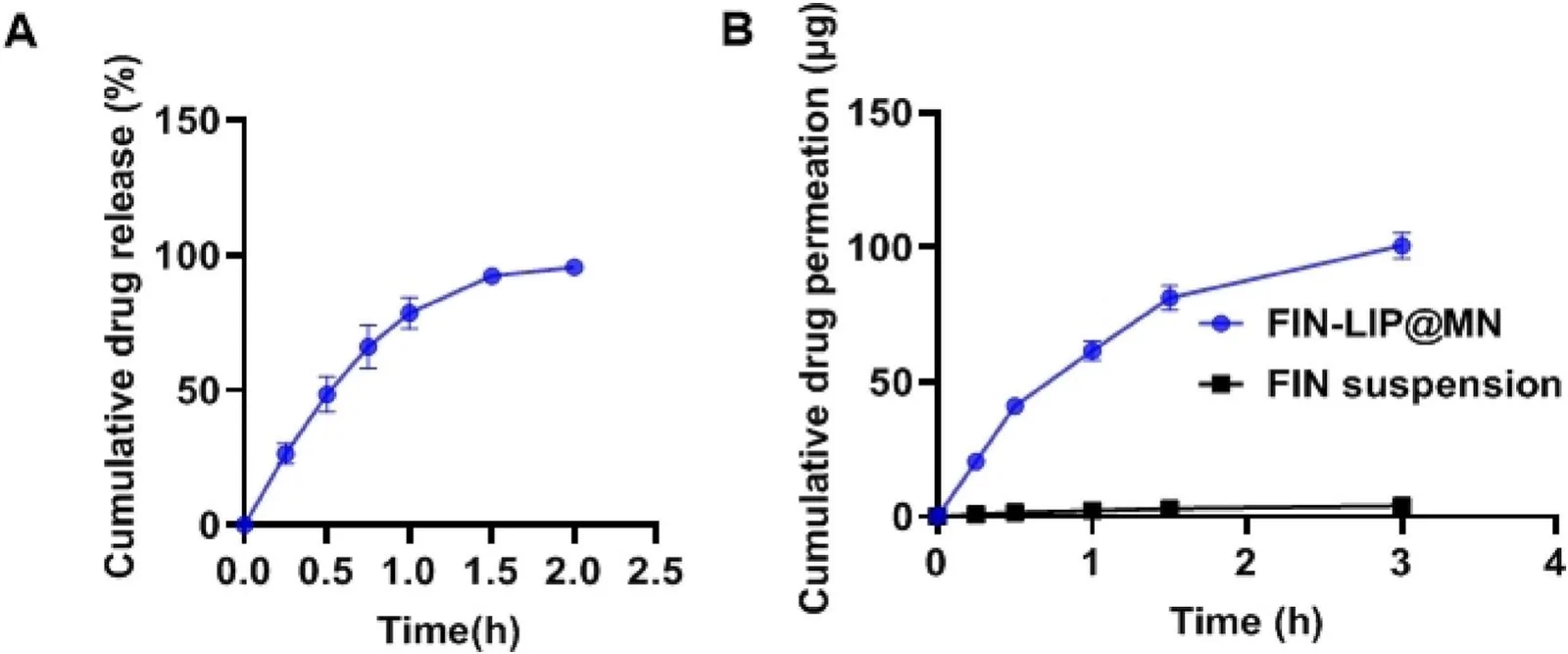
Release and permeation behaviors of FIN-LIP@MNs. (A) Cumulative payload release from FIN-LIP@MNs (*n* = 4); (B) Corneal penetration profiles of FIN formulations (*n* = 4).

**Table 3:** Simulations of drug release from FIN-LIP@MNs.

Simulation model	Release kinetic equation	Correlation coefficient (R^2^)
Zero-Order	$Q_{r}=7.12t$	0.81
First-Order	$Q_{r}=100\;\left(1-e^{-1.61t}\right)$	0.98
Higuchi	$Q_{r}=32.71\;\sqrt{t}$	0.85
Ritger-Peppas	$Q_{r}=71.81\;t^{0.14}$	0.60

#### 3.5.6 Ex vivo transcorneal delivery behavior of FIN-LIP@MNs

The transepithelial permeation kinetics of FIN were investigated using Franz-type diffusion cells (Li et al., 2021). Over the 6 h experimental period, the FIN-LIP@MNs exhibited progressively enhanced drug permeation (Fig. 4B), whereas the unencapsulated suspension showed negligible corneal penetration. Furthermore, the steady-state flux of the MN formulation (17.2 ± 1.2 μg·cm^−2^·h^−1^) markedly exceeded that of the conventional aqueous formulation (1.1 ± 0.8 μg·cm^−2^·h^−1^). Athough nanoparticulate carriers may improve the apparent solubility of lipophilic therapeutics, their relatively large hydrodynamic size frequently limits transepithelial permeation efficiency. By transiently disrupting the corneal epithelium, MNs create micron-scale channels that facilitate accelerated drug transport (Ansari et al., 2025). The marked superiority of FIN-LIP@MNs over conventional formulations suggests enhanced potential for intraocular delivery of the therapeutic agent.

### 3.6 In vivo safety of FIN-LIP@MNs

Mice received MN patch application to the corneal surface for 5 min following systemic anesthesia (Fig. 5A). The reversibility of this microtrauma was assessed using transepidermal water loss (TEWL) quantification and sodium fluorescein staining. Intact corneas exhibited baseline TEWL of 13.3 ± 1.3 g·m^−2^ h^−1^, which rose markedly to 26.9 ± 1.6 g·m^−2^ h^−1^ following MN treatment (Fig. 5B), indicative of transient microchannel generation. Notably, TEWL returned to baseline within 45 min of patch removal, demonstrating the minimally invasive and spontaneously resolving character of MN-induced epithelial disruption. Fluorescein staining, the established gold standard for evaluating corneal epithelial integrity (Li et al., 2021), showed significantly reduced fluorescence at 45 min post-removal, further validating prompt epithelial restoration (Fig. 5C). These results collectively confirm the fully reversible nature of MN-induced corneal microtrauma and establish FIN-LIP@MNs as a safe and effective modality for transcorneal therapeutic delivery.

**Fig. 5:**
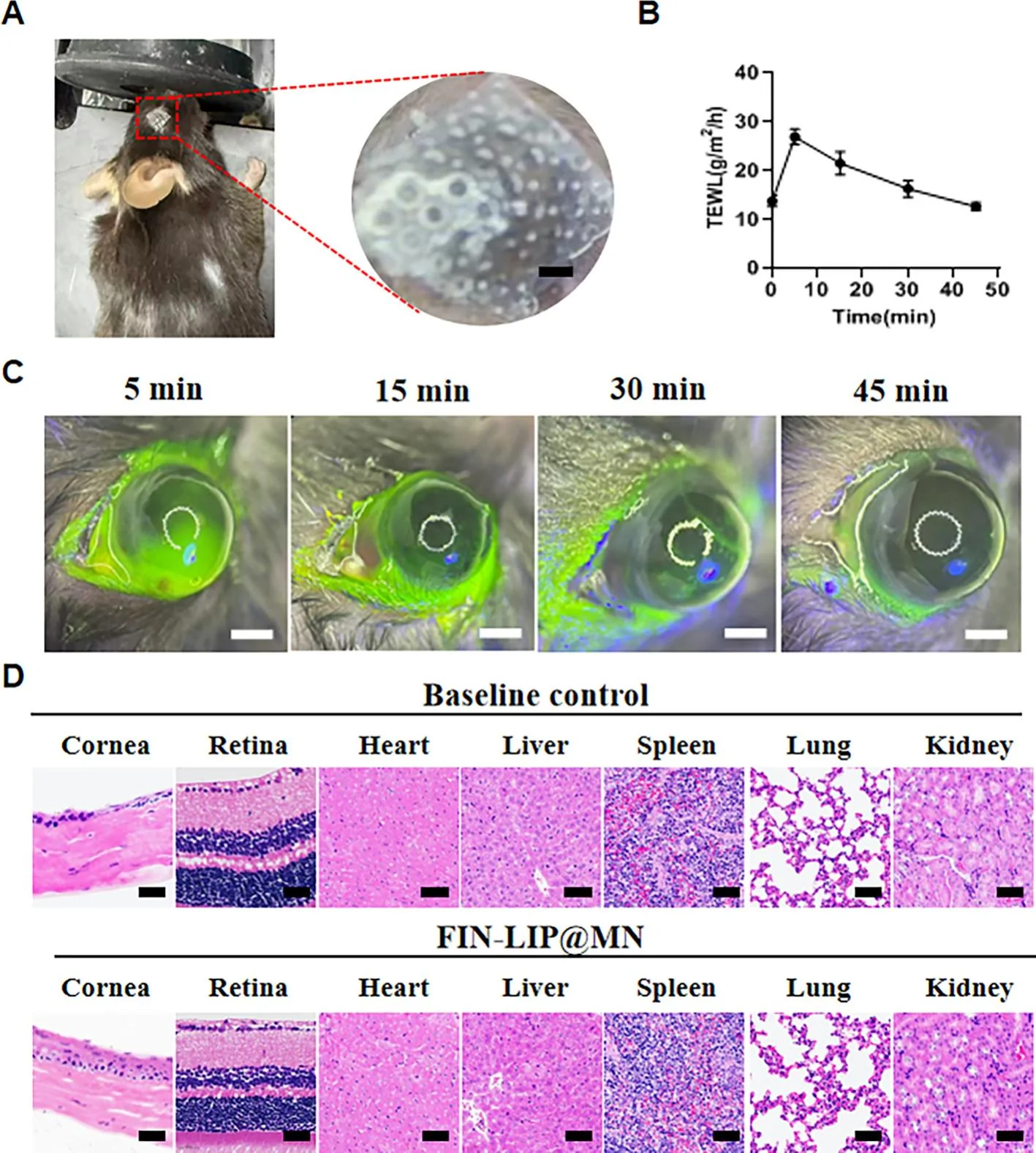
Safety characterization of FIN-LIP@MNs in vivo. (A) Illustrative schematic of MN insertion into the mice cornea; (B) Longitudinal monitoring of corneal epithelial barrier integrity post-administration; (C) Fluorescence images depicting corneal epithelial regeneration dynamics (scale bar = 500 μm); (D) H&E staining of major organs following 7 consecutive days of MN administration (scale bar = 50 μm).

### 3.7 Enhanced anterior and posterior segment drug exposure via FIN-LIP@MNs

To overcome the anatomical and physiological limitations of conventional topical ophthalmic therapy, the transcorneal delivery performance of FIN-LIP@MNs was evaluated. Standard eye drops face significant barriers at the ocular surface, including rapid tear turnover via reflex lacrimation and nasolacrimal drainage, which collectively limit corneal absorption to approximately 5% of the administered dose (Kim et al., 2017). This low bioavailability necessitates frequent administration, thereby reducing therapeutic compliance (Gade et al., 2024). Moreover, the stratified epithelial barrier, comprising apical tight junction complexes and a mucin-rich tear film, presents a substantial obstacle to the permeation of lipophilic agents and macromolecular drugs (Huang et al., 2024; Yang et al., 2023). Ocular MNs overcome these limitations by mechanically permeabilizing the epithelial layer, creating transient microchannels that enable intrastromal drug deposition and direct anterior chamber access without permanent tissue damage (Shi et al., 2022; Yu et al., 2025). A significant enhancement in ocular drug exposure was observed for FIN-LIP@MNs compared with conventional FIN suspensions (Fig. 6; Table 4). The MN formulation achieved a peak aqueous humor concentration of 597.6 ± 68.2 ng/mL, representing a 28.4-fold increase over the suspension group (21.5 ± 5.1 ng/mL, **p = 0.008 < 0.01). Correspondingly, the area under the concentration–time curve (AUC_0-∞_) showed a 26.9-fold increase (4383.6 ± 181.5 versus 162.6 ± 18.1 ng·h/mL, **p = 0.003 < 0.01), indicating substantially improved ocular exposure. Moreover, the mean residence time (MRT_0-∞_) of the FIN-LIP@MN group was extended to 6.3 ± 1.0 h, confirming sustained intraocular retention compared with the transient exposure observed after topical instillation. Thus, FIN-LIP@MNs enable both rapid transcorneal transport and prolonged intratissue drug retention, overcoming the dual limitations of rapid ocular surface clearance and poor permeability characteristic of conventional eye drop formulations.

**Fig. 6:**
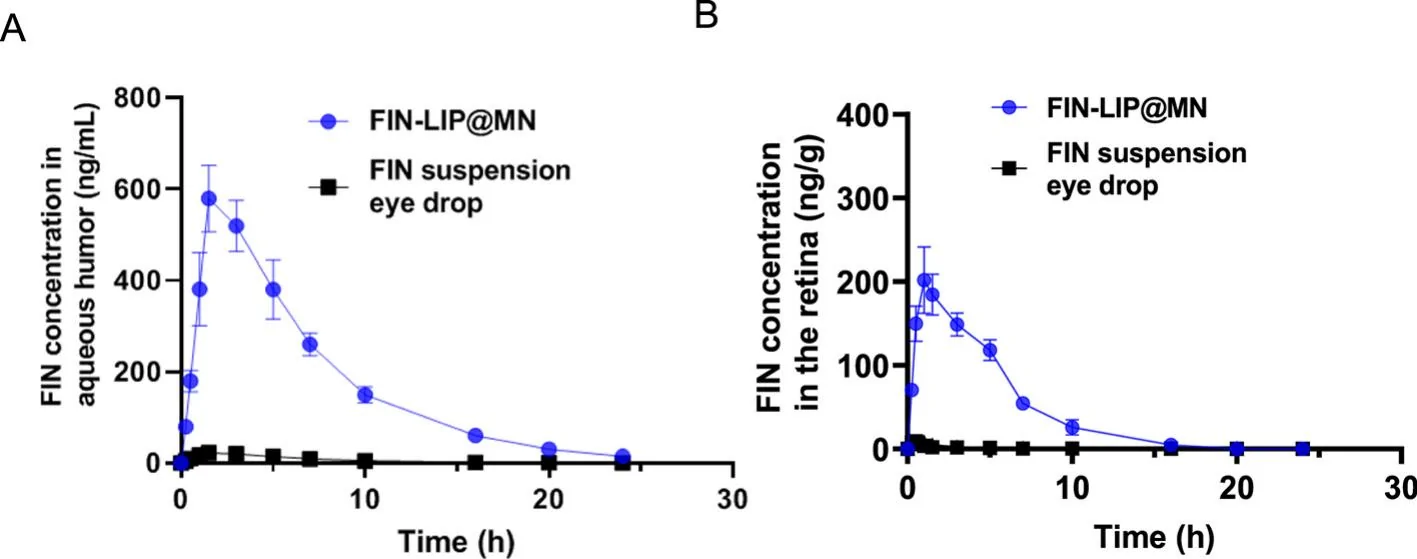
Ocular pharmacokinetic profiles of FIN. (A) Aqueous humor and (B) retinal concentrations following a single application of FIN-LIP@MNs or FIN suspension eye drops (*n* = 6 per time point). Data are presented as mean ± standard deviation (SD).

**Table 4:** Pharmacokinetic parameters of FIN in aqueous humor following administration of different FIN formulations (*n* = 6).

Formulation	Pharmacokinetic parameters			
	C_max_(ng/mL)	T_max_ (h)	AUC_0~∞_ (ng·h/mL)	MRT_0~∞_ (h)
FIN-LIP@MN	597.6 ± 68.2^⁎⁎^	2.0 ± 0.7	4383.6 ± 181.5^⁎⁎^	6.3 ± 1.0^⁎^
FIN suspension	21.5 ± 5.2	1.7 ± 0.5	162.6 ± 18.1	3.1 ± 0.8

Building upon the enhanced anterior chamber exposure, we further quantified FIN concentrations in retinal tissue to assess posterior segment delivery (Fig. 6B; Table 5). The MN formulation achieved a peak retinal concentration (C_max_) of 201.8 ± 39.8 ng/g at approximately 1.0 ± 0.2 h post-application. In stark contrast, the FIN suspension yielded a C_max_ of only 9.0 ± 1.5 ng/g at 0.5 ± 0.1 h, representing a 22.4-fold increase in peak retinal exposure for the microneedle system. More importantly, the AUC_0~∞_ for FIN-LIP@MNs reached 1150.4 ± 79.7 ng·h/g, whereas that of the eye-drop formulation was merely 24.1 ± 1.6 ng·h/g, corresponding to a 47.7-fold improvement in total retinal drug exposure. The prolonged mean residence time (MRT_0~∞_) of 4.5 ± 0.7 h for FIN-LIP@MNs, compared with 1.2 ± 0.3 h for the suspension, further corroborates the efficient drug delivery behavior of FIN-LIP@MNs at the target tissue.

**Table 5:** Pharmacokinetic parameters of FIN in retinal tissue following administration of different FIN formulations (*n* = 6).

Formulation	Pharmacokinetic parameters			
	C_max_(ng/g)	T_max_ (h)	AUC_0~∞_ (ng·h/mL)	MRT_0~∞_ (h)
FIN-LIP@MN	201.8 ± 39.8^⁎⁎^	1.0 ± 0.2	1150.4 ± 79.7^⁎⁎⁎^	4.5 ± 0.7^⁎^
FIN suspension	9.0 ± 1.5	0.5 ± 0.1	24.1 ± 1.6	1.2 ± 0.3

### 3.8 FIN-LIP@MNs provided effective anti-angiogenic intervention

#### 3.8.1 Effective restoration of retinal architecture by FIN-LIP@MNs

Histopathological analysis of retinal coronal sections revealed distinct morphological phenotypes among the experimental groups. In the baseline control group, the retina exhibited an intact ten-layer architecture with densely packed nuclei and clearly defined lamination, including the nerve fiber layer (NFL), ganglion cell layer (GCL), inner plexiform layer (IPL), inner nuclear layer (INL), outer plexiform layer (OPL), outer nuclear layer (ONL), and photoreceptor segments (Fig. 7A). In the untreated control (DR model) and blank MN groups, severe microvascular and neurodegenerative lesions were observed, including prominent microaneurysms at the NFL–GCL junction, marked retinal edema characterized by interlaminar clefting and spongiform loosening between the GCL and INL, and significantly reduced ganglion cell density with loss of orderly stratification. These findings indicate that the dissolvable MN matrix alone, without a pharmacological payload, provides no meaningful therapeutic benefit. The FIN suspension group showed partial attenuation of these pathological lesions, whereas the FIN-LIP@MN group achieved near-complete resolution of both interlaminar edema and microaneurysms, accompanied by marked restoration of ganglion cell density and re-establishment of orderly retinal stratification. Collectively, these histopathological findings demonstrate that FIN-LIP@MN-mediated transcorneal delivery provides robust neurovascular protection and effectively alleviates pathological retinal alterations associated with diabetic retinopathy.

**Fig. 7:**
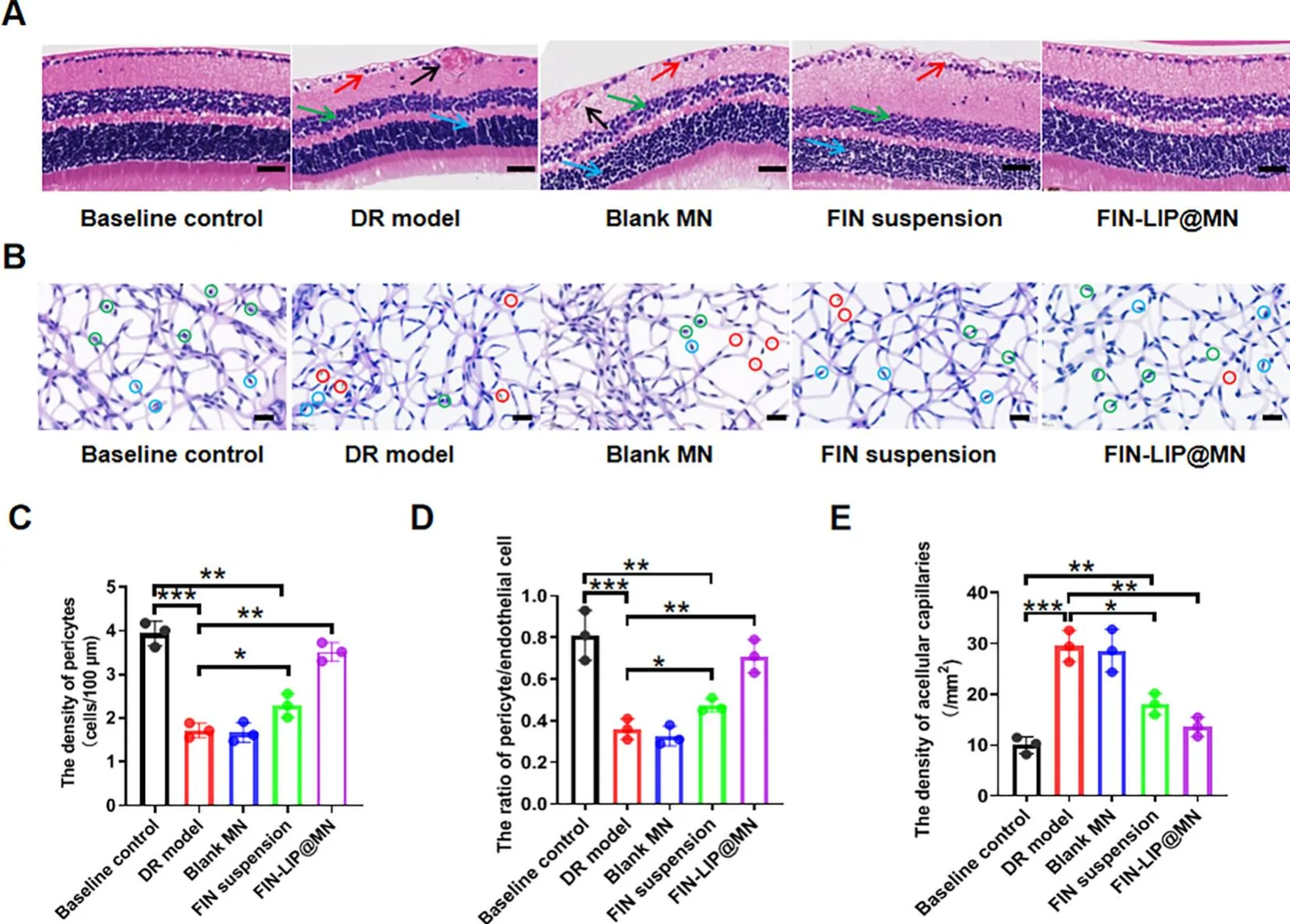
FIN-LIP@MNs rescued retinal microvascular integrity in db/db mice. (A) Representative H&*E*-stained retinal coronal sections illustrating retinal laminar architecture across all groups; Red arrows indicateganglion cells, black arrows denote microaneurysms, and green and blue arrows mark disorganized inner nuclear layers and outer plexiform layers (scale bar = 50 μm); (B) PAS staining of retinal vasculature with pericytes (green), endothelial cells (blue), and acellular capillaries (red) indicated (scale bar = 50 μm); (C) Quantification of pericyte density (cells/100 μm); (D) Quantification of the pericyte-to-endothelial cell (P/E) ratio; (E) Quantification of acellular capillary density (/mm^2^). Data are presented as mean ± SD (*n* = 3), *p < 0.05, **p < 0.01, ***p < 0.001. (For interpretation of the references to colour in this figure legend, the reader is referred to the web version of this article.)

#### 3.8.2 Effective restoration of of pericyte–endothelial homeostasis by FIN-LIP@MNs

Periodic acid-Schiff (PAS) staining of retinal flat mounts revealed distinct microvascular phenotypes among the experimental groups (Fig. 7B, C, D, and E). The baseline control group exhibited a regular polygonal capillary network with densely distributed pericytes, yielding a pericyte-to-endothelial cell (P/E) ratio of 0.8 ± 0.1 and a low acellular capillary density of 9.8 ± 1.1/mm^2^. In contrast, the DR model group displayed severe pericyte loss (1.7 ± 0.2 cells/100 μm, **p = 0.007 < 0.01 vs. healthy control), a markedly reduced P/E ratio (0.4 ± 0.1, **p = 0.008 < 0.01 vs. healthy control), and abundant acellular capillaries (29.5 ± 3.1/mm^2^, **p = 0.004 < 0.01 vs. healthy control). The blank MN group exhibited comparable pathological features (pericyte density: 1.7 ± 0.2 cells/100 μm, P/E ratio: 0.4 ± 0.06, acellular capillaries: 28.7 ± 2.9/mm^2^; all *p* > 0.05 vs. DR model), confirming that microneedle insertion alone conferred no therapeutic benefit. The FIN suspension group showed partial improvement (pericyte density: 2.3 ± 0.3 cells/100 μm, *p = 0.03 < 0.05 vs. DR model; P/E ratio: 0.5 ± 0.05,*p = 0.04 < 0.05 vs. DR model; acellular capillaries: 18.1 ± 2.1/mm^2^, *p = 0.04 < 0.05 < 0.01 vs. DR model), yet remained significantly inferior to the baseline control group (pericyte density: *p = 0.04 < 0.05; P/E ratio: *p = 0.04 < 0.05; acellular capillaries: *p = 0.03 < 0.05), attributable to limited corneal penetration. In contrast, the FIN-LIP@MN group achieved near-complete restoration, with pericyte density (3.5 ± 0.2 cells/100 μm, *p =* 0.7 > 0.05 vs. baseline control) and P/E ratio (0.7 ± 0.06, *p =* 0.8 > 0.05 vs. healthy control) recovering to levels statistically comparable to those of the healthy control group, while acellular capillary density decreased to 13.6 ± 1.8/mm^2^ (*p = 0.02 < 0.05 vs. DR model; *p* = 0.7 > 0.05 vs. healthy control), representing a 53.8% reduction compared to the DR model group.

The therapeutic superiority of FIN-LIP@MNs is attributed to the synergistic integration of lipid encapsulation, which overcomes the poor aqueous solubility of FIN, and MN-mediated intrastromal delivery, which bypasses the corneal epithelium to achieve sustained retinal drug exposure and restore the physiological P/E ratio.

#### 3.8.3 Highly efficient downregulation of retinal CD31 expression by FIN-LIP@MNs

Retinal neovascularization, a hallmark of proliferative diabetic retinopathy, is characterized by pathological endothelial cell proliferation and migration, with CD31 serving as a representative marker of activated vascular endothelium (Chavkin et al., 2021). To evaluate the anti-angiogenic efficacy of FIN-LIP@MNs at the molecular level, immunofluorescence staining of retinal CD31 was performed in db/db mice at week 12 following diabetes induction. In the baseline control group, retinal vascular architecture remained intact, and CD31 expression was maintained at a low basal level (Fig. 8A). In contrast, the DR model group exhibited markedly increased CD31-positive staining, accompanied by dense and disorganized retinal vasculature, indicating abnormal endothelial cell proliferation and enhanced angiogenesis in the diabetic retina (Fig. 8A and B). This finding is consistent with the established pathophysiology of diabetic retinopathy, in which chronic hyperglycemia-induced hypoxia and inflammation activate VEGF-mediated angiogenic signaling cascades. Moreover, treatment with blank MNs produced negligible reduction in CD31 fluorescence intensity compared with the diabetic control group, suggesting that mechanical insertion alone and the liposomal excipients exerted minimal anti-angiogenic effects. This observation highlights the necessity of an active drug payload for therapeutic efficacy rather than simple physical intervention.

**Fig. 8:**
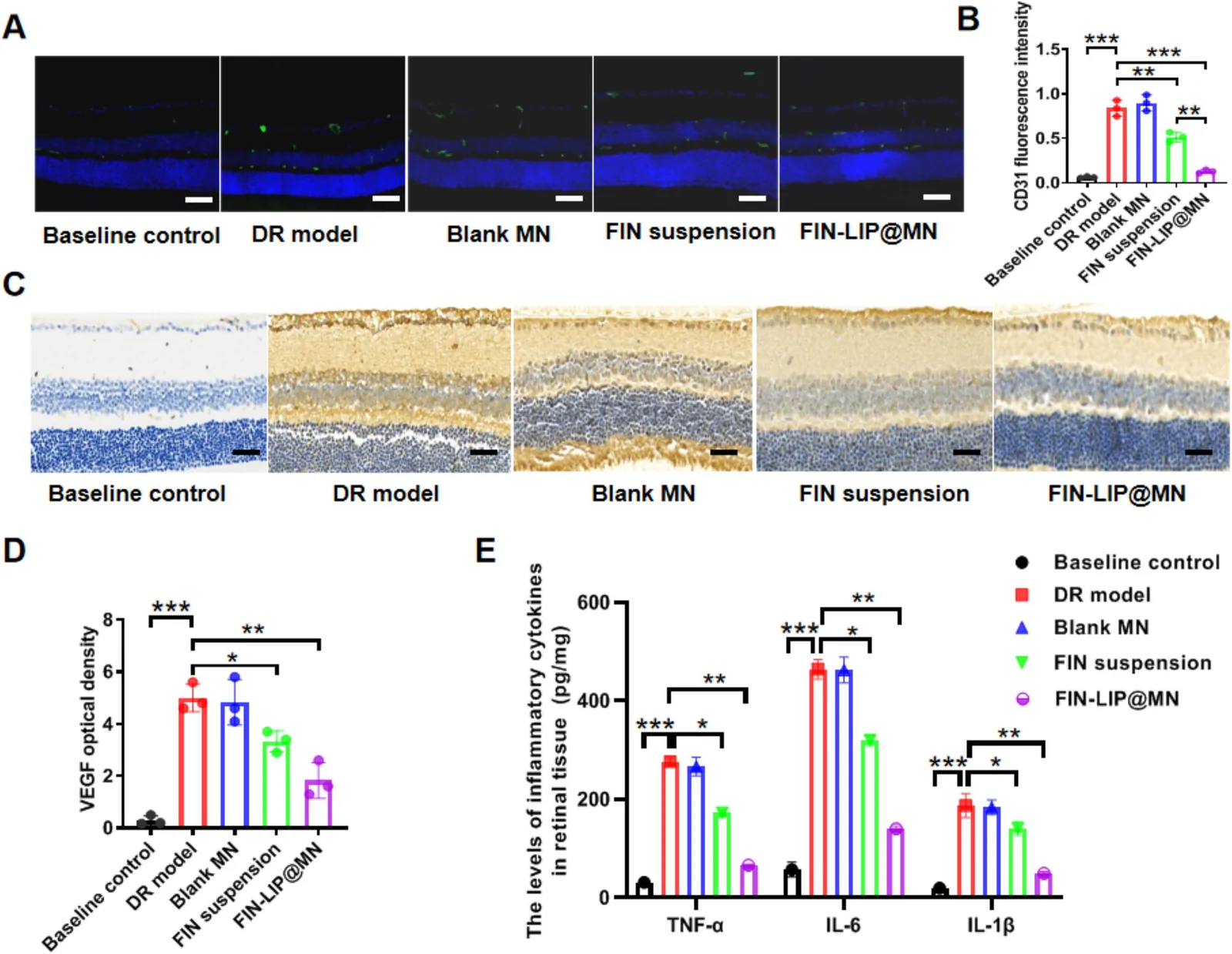
Therapeutic efficacy of FIN-LIP@MNs in suppressing diabetic retinopathy-associated angiogenesis and neuroinflammation. (A) Representative immunofluorescence micrographs depicting CD31 expression in retinal tissue (green, scale bar = 50 μm); (B) Semiquantitative analysis of CD31 mean fluorescence intensity across all groups (*n* = 3); (C) Representative immunohistochemical images of VEGF expression in retinal sections (scale bar = 50 μm); (D) Quantitative analysis of VEGF mean optical density across all groups (*n* = 3); (E) Retinal levels of pro-inflammatory mediators (TNF-α, IL-6, and IL-1β) determined by ELISA (*n* = 3). Data are presented as mean ± SD, ***p < 0.001, **p < 0.01, *p < 0.05. (For interpretation of the references to colour in this figure legend, the reader is referred to the web version of this article.)

Topical administration of FIN suspension resulted in moderate downregulation of CD31 expression. However, CD31-positive staining remained evident in the mid-peripheral retina, with fluorescence intensity reduced by only 28.3 ± 4.7% compared with the DR model group (**p = 0.006 < 0.01). This limited efficacy may be attributed to the poor corneal bioavailability (<2%) and rapid precorneal clearance associated with conventional eye drop formulations. In contrast, the FIN-LIP@MN group exhibited markedly reduced CD31 immunoreactivity, with sparse and discontinuous fluorescent signals primarily localized to the major retinal vessels, and a significant 76.8 ± 5.2% reduction in CD31-positive fluorescence intensity compared with the DR model group (***p = 0.0004 < 0.001). This anti-angiogenic effect significantly exceeded that observed in the FIN suspension group (**p = 0.003 < 0.01). The superior suppression of pathological neovascularization achieved by FIN-LIP@MNs suggests successful delivery of therapeutically effective FIN concentrations to the retinal microenvironment.

#### 3.8.4 Effective suppression of retinal VEGF expression by FIN-LIP@MNs

Vascular endothelial growth factor (VEGF) serves as a key regulator of pathological angiogenesis in diabetic retinopathy by promoting endothelial cell proliferation, migration, and vascular permeability (Lennikov et al., 2025; Zhang et al., 2023). The results demonstrated that VEGF expression in the DR model group was significantly upregulated compared with that in the baseline control group, reaching approximately 14-fold higher levels (***p **=** 0.0009 < 0.001, Fig. 8C and D). Blank MNs showed no significant effect on VEGF protein expression compared with the DR model group (*p =* 0.7 > 0.05), further confirming that mechanical insertion alone exerts minimal anti-angiogenic activity (Fig. 8C and D). Moreover, topical administration of FIN suspension resulted in moderate downregulation of VEGF expression (39.6 ± 2.9% reduction, vs. DR model group, *p = 0.01 < 0.05), consistent with its limited corneal penetration and rapid precorneal clearance (Fig. 8C and D). Most importantly, FIN-LIP@MNs markedly reduced VEGF immunoreactivity, decreasing VEGF-positive staining intensity by 64.1 ± 5.8% compared with the DR model group (**p = 0.004 < 0.01). This anti-angiogenic efficacy substantially exceeded that observed in both the FIN suspension and blank MN groups. Overall, the potent suppression of VEGF by FIN-LIP@MNs can be attributed to the synergistic integration of liposomal encapsulation and MN-mediated delivery.

#### 3.8.5 Anti-Inflammatory efficacy of FIN-LIP@MNs in DR

Building upon the robust suppression of retinal CD31 and VEGF expression described above, we further evaluated whether FIN-LIP@MNs could simultaneously attenuate the inflammatory microenvironment that perpetuates neovascularization in diabetic retinopathy. Chronic low-grade inflammation is a key pathological hallmark of DR, with TNF-α, IL-6, and IL-1β contributing to neurovascular damage through sustained NF-κB activation (Du et al., 2025). Quantitative ELISA analysis revealed distinct anti-inflammatory effects among the treatment groups (Fig. 8E). The DR model group exhibited marked upregulation of retinal TNF-α (276.1 ± 8.5 pg/mg), IL-6 (464.5 ± 19.7 pg/mg), and IL-1β (187.2 ± 24.5 pg/mg) compared with the baseline control group (TNF-α: 30.6 ± 5.2 pg/mg, ***p **=** 0.0008 < 0.001; IL-6: 56.7 ± 7.9 pg/mg, ***p = 0.0005 < 0.001; IL-1β: 19.2 ± 1.7 pg/mg,***p = 0.0001 < 0.001), confirming the establishment of a chronic pro-inflammatory milieu (Fig. 8E). Blank MNs produced cytokine levels comparable to those of the DR control group (all *p* > 0.05), whereas FIN suspension achieved moderate suppression (TNF-α: 37.6%, *p = 0.03 < 0.05; IL-6: 30.2%, *p = 0.02 < 0.05; IL-1β: 26.1%; *p = 0.04 < 0.05). In contrast, FIN-LIP@MNs demonstrated substantial anti-inflammatory efficacy, with reductions of 76.2%, 69.8%, and 71.6% in TNF-α, IL-6, and IL-1β levels, respectively, compared with the DR control group (TNF-α, **p = 0.003 < 0.01; IL-6, **p = 0.005 < 0.01; IL-1β, **p = 0.009 < 0.01). Collectively, these findings indicate that FIN-LIP@MNs function as a dual-action platform that simultaneously targets vascular pathology and the associated inflammatory microenvironment, thereby providing a minimally invasive therapeutic strategy that may reduce the need for systemic corticosteroids or intraocular anti-VEGF injections in the management of diabetic retinopathy.

The first-order release kinetics is mechanistically relevant to the FIN-LIP@MN system. The rapid bioresorption of the PVP K90 matrix achieves complete dissolution within 15 min in corneal tissue, effectively decoupling the microneedle dissolution from the subsequent drug release process. This rapid dissolution eliminates the polymeric barrier, rendering liposome-mediated diffusion the rate-limiting step. Once the MN matrix dissolves, the encapsulated FIN is released from the liposomes into the surrounding tissue following first-order kinetics, wherein the release rate is proportional to the remaining drug concentration within the liposomal bilayer. This concentration-dependent release is characteristic of diffusion-controlled release from lipid vesicles. Clinically, this translates into a favorable biphasic profile. An initial burst release of approximately 52% within 0.5 h facilitates rapid attainment of therapeutic drug concentrations, while the subsequent sustained release ensures prolonged intraocular exposure. This biphasic profile aligns with the observed pharmacokinetic enhancement, as well as the robust therapeutic efficacy observed in the diabetic retinopathy model.

## 4 Conclusion

This study reports the rational design, systematic optimization, and comprehensive preclinical evaluation of FIN-LIP@MNs for the transcorneal treatment of diabetic retinopathy. The optimized FIN-LIP@MNs exhibited sufficient mechanical strength for corneal insertion, rapid bioresorption, and sustained first-order release kinetics. Moreover, FIN-LIP@MNs markedly improved ocular bioavailability, increasing aqueous humor C_max_ and AUC_0~∞_ by 28.4- and 26.9-fold and retinal exposure by 22.4- and 47.7-fold versus conventional eye drops. In a validated DR model, FIN-LIP@MNs achieved near-complete restoration of retinal microvascular architecture, as evidenced by normalization of pericyte density and the pericyte-to-endothelial cell ratio, substantial regression of acellular capillaries, and marked attenuation of pathological angiogenesis and inflammation. FIN-LIP@MNs therefore represent a promising minimally invasive platform capable of simultaneously overcoming drug solubility limitations and corneal barrier restrictions to inhibit the progression of diabetic retinopathy.

## CRediT authorship contribution statement

**Weili Zhang:** Writing – original draft, Methodology. **Lu Wang:** Methodology. **Wen Qin:** Methodology. **Yingrong Chen:** Formal analysis. **Shenyun Fang:** Writing – review & editing. **Zhenchao Ma:** Supervision. **Tao Xue:** Writing – review & editing. **Xiang Yu:** Writing – original draft, Conceptualization.

## Ethics declaration

This study was conducted in accordance with the ARRIVE (Animal Research: Reporting of In Vivo Experiments) guidelines. This study was approved by the Ethical Committee of Huzhou Central Hospital's Academy. (Approval No. 202604004).

## Funding

This work was supported by the Public Welfare Technology Application Research Program of Huzhou (No. 2024GY08 and 2025GYB62), and the General Program of Health Commission of Zhejiang Province (No. 2025KY326),

## Declaration of competing interest

The authors declare that they have no known competing financial interests or personal relationships that could have appeared to influence the work reported in this paper.

## Data Availability

Data will be made available on request.

## Appendix A Supplementary data

Supplementary material: Stability of FIN-LIPs in MN formulations.
