# Supplementary material for: Finerenone liposome-embedded dissolving microneedles: A transcorneal platform for minimally invasive therapy of diabetic retinopathy
Source: Int J Pharm X. 2026 Oct 13;12:100682. doi: 10.1016/j.ijpx.2026.100682 (PMC13691493; doi:10.1016/j.ijpx.2026.100682)
Supplement: Supplementary file 1 [file mmc1.docx]

**Supplemental Material**

**Finerenone Liposome-embedded Dissolving Microneedles: A Transcorneal Platform for Minimally Invasive Therapy of Diabetic Retinopathy**

Weii Zhang ^a, 1^, Lu Wang ^a, 1^, Wen Qin ^b, 1^, Yingrong Chen ^c^, Shenyun Fang ^a, 1^, Zhenchao Ma ^c, *^, Tao Xue ^a,*^, Xiang Yu ^b, c,*^.

*^a^ First Affiliated Hospital of Huzhou Normal University, Huzhou 313000, Zhejiang, China;*

*^b^ College of Pharmacy, Shenzhen Technology University, Shenzhen 518118, China;*

*^c^ Affiliated Huzhou Central Hospital, Huzhou University, Huzhou 313000, Zhejiang, China;*

**^*^**Corresponding author.

*E-mail:* mazhenchao1986@163.com (Zhenchao Ma); xuehutc@163.com (Tao Xue); yuxiang1990@vip.qq.com (Xiang Yu).

^1^ These authors contributed equally to this work.

1. **Stability of FIN-LIPs in MN formulations**

The liposome-loaded microneedles were placed in sealed containers and stored in a stability chamber maintained at 25 ± 2 °C and 20 ± 5% relative humidity (RH). At predetermined time points (0, 3, and 6 months), microneedle patches were randomly withdrawn for analysis. Each patch was dissolved in 20 mL of deionized water at room temperature under gentle agitation to fully reconstitute the liposomal dispersion. The morphology of the recovered liposomes was examined by TEM. For the determination of drug encapsulation efficiency (EE%), the reconstituted liposomal suspension was subjected to ultrafiltration using an Amicon Ultra centrifugal filter (MWCO 10 kDa) at 4,000 × g for 20 min to separate free drug from liposome-encapsulated drug. The free drug content in the filtrate and the total drug content (obtained after disrupting the liposomal membrane with methanol ) were quantified by described in Section 2.4.1. All experiments were performed in triplicate, and the results are expressed as mean ± standard deviation (SD).

The physicochemical stability of FIN-LIPs within the microneedle matrix was maintained throughout the 6-month storage duration. The reconstituted liposomes exhibited intact morphology and consistent particle sizes of approximately 128 nm (Fig. S1A), while encapsulation efficiencies at 0, 3, and 6 months were 79.33 ± 5.52%, 85.67 ± 3.29%, and 78.93 ± 5.87%, respectively. No statistically significant differences were observed across the tested time points (*p* > 0.05, Fig. S1B), confirming that the microneedle formulation effectively stabilizes FIN-LIPs.

**Figure S1 Stability of FIN-LIPs in MN formulations.** (A) Representative TEM micrographs of FIN-LIPs reconstituted from microneedle patches at predetermined time points (0, 3, and 6 months); (B) Drug encapsulation efficiency of FIN-LIPs during 6-month storage at 25 ± 2 °C and 20 ± 5% RH. Data are expressed as mean ± SD (n = 3).
